# Harnessing combined p19Arf and interferon-beta gene transfer as an inducer of immunogenic cell death and mediator of cancer immunotherapy

**DOI:** 10.1038/cddis.2017.201

**Published:** 2017-05-11

**Authors:** Aline Hunger, Ruan FV Medrano, Bryan E Strauss

**Affiliations:** 1 Viral Vector Laboratory, Center for Translational Investigation in Oncology, Cancer Institute of Sao Paulo/LIM 24, University of Sao Paulo School of Medicine, Sao Paulo, Brazil

Cancer immunotherapy is a wide-ranging term that includes many strategies to reestablish or activate an effector antitumor immunity cycle. Among the most successful approaches, PD-1/PDL-1 and CTLA-4 checkpoint blockade and chimeric antigen receptor (CAR) T cells have provided undeniable evidence of the potential of immunotherapy for several types of cancer, including metastatic melanoma.^1^

Another promising approach that aims to provide both antigenic and adjuvant signals in order to initiate an adaptive immune response is the induction of immunogenic cell death (ICD). It was originally described as a molecular response of cancer cells to treatment with chemotherapeutic agents (e.g., anthracyclines), and involves the orchestrated release of the danger signals calreticulin, ATP and HMGB-1.^2^ Interestingly, this cell death process can be triggered by viral infections and is one of the mechanisms by which some oncolytic viruses, such as those based on herpes simplex virus type 1 (HSV-1), induce an immune response.^3^ However, to the best of our knowledge, gene transfer methods mediated by nonreplicative viral vectors, such as adenovirus, have yet not been described as inducers of ICD.

With the objective of reestablishing both intrinsic cell death mechanisms and cancer immune surveillance, our lab developed a unique set of adenoviral vectors for the gene transfer of both p19Arf (functional partner of p53) and interferon-*β* (IFN*β*, immunomodulatory cytokine). As elucidated in our recent work,^4^ targeting the p19Arf and IFN*β* pathways created interplay between (i) p53/Arf pro-apoptotic signaling, (ii) the adenovirus delivery vehicle and (iii) an IFN*β* antiviral/immunostimulatory pathway, culminating in a cell death process that displays features of necroptosis and provides an ICD stimulus to the adaptive immune system compartment (Figure 1).

Mechanistically speaking, since melanomas often retain wild-type p53, we reasoned that this powerful tumor suppressor could be recruited to assist in the treatment and also promote high levels of transgene expression from our adenoviral vector, which employs a synthetic p53-responsive promoter called PGTx*β*. In fact, we have shown that the p53-responsive promoter outperforms the typically employed cytomegalovirus (CMV) immediate-early promoter or retroviral long terminal repeat (LTR), providing 5−7 times higher transgene expression.^5^ In our current work, an additional improvement was made to this nonreplicating, serotype 5 adenoviral vector platform, the use of a modified adenoviral fiber protein containing the RGD tripeptide. With this alteration, the adenoviral vector no longer depends on the CAR for entry and instead interacts with integrins. Thus, the viral vector developed for this study offers robust transgene expression as well as ample tropism.

Cooperation between the p53/Arf and IFN pathways has been reported previously, showing that type I IFN’s antiviral defense is enhanced by p53 activity, and that type I IFNs can activate p53 at the transcriptional and post-translational levels.^6, 7, 8^ We expect that p19Arf+IFN*β* should cooperate to activate p53, promote expression from the viral vector due to the p53-responsive promoter, bring about cell death and activate the immune system.

Leading up to this study, we were the first to show that combined, but not individual, p19Arf+IFN*β* gene transfer enhanced killing of B16 mouse melanoma cells *in vitro* and *in vivo*.^9^ We had also shown several aspects of an antitumor immune response, mediated by natural killer cells, CD4+ and CD8+ T lymphocytes, that occurred only when combined gene transfer was applied in prophylactic and therapeutic vaccine models or in *in situ* gene therapy of primary tumors.^10, 11^ However, we did not have an in-depth understanding of the tumor cell’s molecular response to gene transfer nor had the cell death mechanism been thoroughly explored.

Among our recent findings,^4^ we show that p19Arf supplied by adenovirus-mediated gene transfer sensitized B16 cells to the effects of IFN*β* secreted by neighboring cells. That is, IFN*β*’s bystander effect was enhanced when p19Arf was present. We next explored the importance of gene transfer as compared to the use of drugs for the stimulation of the p53/Arf and IFN pathways. While Nutlin-3 (which, similar to p19Arf, frees p53 from MDM2) could substitute p19Arf gene transfer, and Poly (I:C) or recombinant IFN*β* protein could replace the vector encoding IFN*β*, the presence of the adenoviral vectors was necessary for the induction of high levels of cell death. Interestingly, Poly (I:C)+Nutlin-3 induced significant cell killing only in the presence of an innocuous adenoviral vector encoding eGFP, suggesting involvement of an antiviral response. Indeed, we confirmed the induction of genes associated with antiviral response, including Dram1, Chop, Nlrc5 and ISG15, especially so in the presence of combined p19Arf+IFN*β* gene transfer, both *in vitro* and *in vivo*.

While markers of apoptotic cell death (AnnexinV staining, upregulation of Bax and caspase 3 activity), were significantly enhanced when cells were treated with just p19Arf, addition of IFN*β* to p19Arf treatment altered the cell death as compared to the transfer of p19Arf alone. Strikingly, inhibition of pan-caspase activity with Z-VAD-FMK drastically increased cell killing upon p19Arf of IFN*β* single-gene transfer and had no effect on the p19Arf+IFN*β* combination, thus suggesting that alternate routes of cell death were involved. In fact, RIP3, a key mediator of necroptosis, and Tnfrsf1A, an activator of the necrosome complex, were specifically induced upon combined p19Arf and IFN*β* gene transfer, indicating necroptosis as a possible mechanism of cell death. Also, the induction of all three classic ICD markers (calreticulin exposure, ATP secretion and HMGB1 release) was seen only upon combined gene transfer, in agreement with recent findings showing that necroptotic cells undergo ICD upon chemotherapy treatment.^12^

Moreover, as revealed by microarray analysis, cooperation between the p53/Arf and IFN*β* pathways in the context of adenoviral transduction resulted in the induction of an antiviral response. Remarkably, only p19Arf+IFN*β* treatment induced gene expression signatures related to the p53 signaling pathway and apoptosis as well as immune response, response to virus and antigen processing. This may explain why high levels of cell death in addition to release of immunogenic markers were only seen by this combined treatment. Also, all of these treatments were able to inhibit expression of genes related to cell cycle function.

The data described here provide a molecular framework that supports the successful immunotherapy described in our previous studies where vaccines or *in situ* gene therapy with p19Arf+IFN*β* could reduce the progression of challenge tumors.^10, 11^ We propose that our approach may provide advantages not seen with existing immunotherapies. For example, gene transfer is not associated with strong adverse reactions, such as the cardiotoxicity seen with a bona fide ICD inducer like doxorubicin. Since we do not employ an oncolytic vector, impedance of virus spread due to the immune response is not a concern.

Even with the advances detailed in our study, additional points remain unexplored. Some authors suggest that prolonged binding to Tnfrsf1A may result in exacerbated TAK1 activation, which in turn results in RIP3 phosphorylation and activation.^13^ We do not discard the possibility that DAI/ZBP1, the DNA-dependent activator of interferon regulatory factors, may be involved in the RIP1-independent induction of RIP3, since the presence of adenovirus, a possible source of cytoplasmic dsDNA, was important for the induction of high levels of cell death. However, the involvement of TAK1 and DAI in our immunotherapy remains to be determined experimentally.

Certainly, further development is necessary before we can suggest the application of our approach in the clinical setting. Even so, the work described here was critical for exposing the advantages that combined p19Arf and IFN*β* gene transfer brings to the cancer immunotherapy arena.

## Figures and Tables

**Figure 1:**
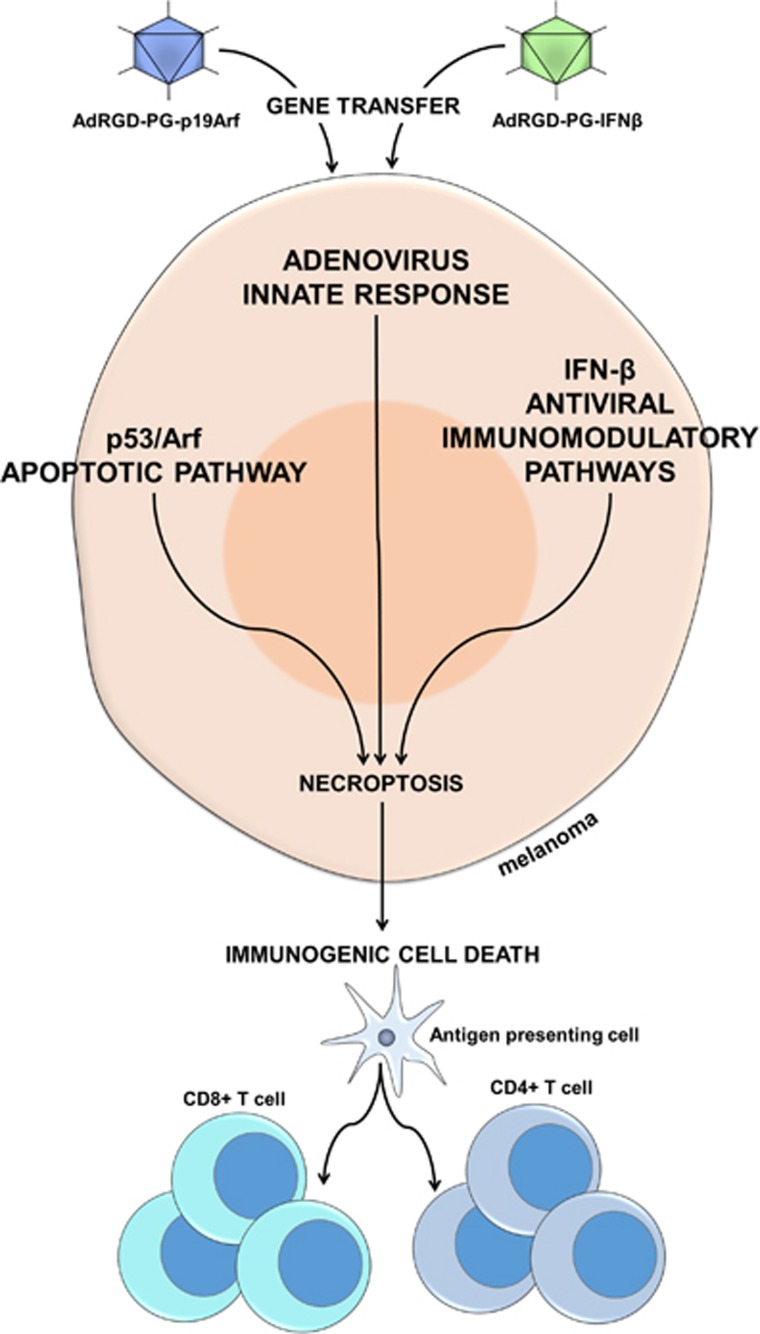
Proposed model for the mechanisms culminating in cell death and immune activation upon p19Arf and interferon-*β* gene transfer. Initially, on remediation of p19Arf by the AdRGD-PGp19Arf adenoviral vector, p53 becomes free-form MDM2 and activates its pro-apoptotic pathway, evidenced by upregulation of its target genes, caspase-3 activity and Bax protein levels. Just as Arf is not strong enough to cause massive cell death on its own, IFN*β* by itself mainly inhibits proliferation and potentiates an antiviral and immunostimulatory response, facilitated by the presence of adenovirus components. Combined activation of these pathways provides a stimulus strong enough for the efficient killing of melanoma cells. This process of cell death displays features of necroptosis, suggested by RIP3 expression, upregulation of the TNF receptor, absence of caspase-3 activity and most importantly, immunogenic cell death markers (ATP, calreticulin, HMGB1), along with IFN*β*, to promote an antitumor immune response mediated by NK cells, CD4+ and CD8+ T lymphocytes
